# Tackling the Unyielding Challenge of Necrotic Unresectable Hepatocellular Carcinoma: A Liver Necrosectomy Approach for Intratumoral Hemorrhage and Abscess Resolution

**DOI:** 10.7759/cureus.58057

**Published:** 2024-04-11

**Authors:** Alejandro Martinez-Esteban, Cielo S Silva-Ramos, Natalia M Barron-Cervantes, Victor J Visag-Castillo

**Affiliations:** 1 General and Gastrointestinal Surgery Service, Fundación Clínica Medica Sur, Mexico City, MEX

**Keywords:** hepatocarcinoma necrosis, liver hemorrhage, liver abscess, hepatocellular carcinoma, liver necrosectomy

## Abstract

Hepatocellular carcinoma (HCC) is one of the most common causes of gastrointestinal and hepatobiliary cancer worldwide. Chronic liver disease and cirrhosis persist as the most common risk factors, typically linked to instances of alcohol abuse or viral infections, notably hepatitis B and hepatitis C infection. Diagnosis can be made using patient history and image studies as there is no need for pathological confirmation. The only curative treatment is surgical resection, and in cases where the tumor is unresectable, as the one presented in this case, and when there are no contraindications, the only option is an orthotopic liver transplantation. This malignancy is not only associated with high mortality but also high morbidity associated with severe complications, such as hemorrhage, necrosis, and infection of the tumor. The significant relevance of this case lies in its capacity to illustrate that despite remaining in non-surgical management for months when an acute complication presented, it was timely identified and surgically treated. The emergence of complications, such as necrosis accompanied by abscess formation and intratumoral hemorrhage, represents an indication for prompt surgical management.

## Introduction

Hepatocellular carcinoma (HCC) is the most common gastrointestinal and hepatobiliary cancer, the primary liver malignancy, and the ninth most common cause of death in the United States, being the leading cause of cancer-related death in the world [1]; its incidence is higher in men, presenting a relation 2.4:1, and generally follows a geographical distribution with hepatitis B and C virus, especially in South-eastern Asia, Middle-west Africa, Melanesia, and Polynesia [2], The worldwide age-standardized annual mortality rates of liver cancer are 13.9 per 100,000 in men and 4.9 per 100,000 in women [2,3]. The most important risk factors for HCC are preexisting liver cirrhosis and hepatitis B infection (due to both the direct oncogenic effect and risk of cirrhosis). Risk factors for liver cirrhosis (and therefore risk factors for HCC) include hepatitis C infection, alcohol use, and non-alcoholic steatohepatitis [4]. Chronic liver disease is the principal risk factor; it is important to mention that HCC typically manifests in individuals who have been afflicted with cirrhosis over a prolonged duration. The most common cause of chronic hepatitis is a viral infection, HBV and HCV being the most commonly presented; however, several epidemiological studies have proven that the most significant cause is chronic HCV infection [3]. HBV carriers have a 10 to 25% lifetime risk of presenting HCC at some point in their lives. An active infection, defined by HBV DNA levels >105/mL, is associated with a 2.5 to 3 times increased risk of developing HCC in eight to ten years of follow-up [5]. Both hepatitis B surface antigen (HBsAg) and hepatitis B core antibody (anti-HBc) are useful in the diagnostic approach. It is also important to emphasize that timely treatment that allows viral suppression significantly reduces the risk of presenting HCC from HBV [3]. 

Other important causes that have increased in prevalence and incidence in recent years are alcohol-related cirrhosis and non-alcoholic fatty liver disease (NAFLD) [3]. Not only is HCC associated with a high mortality rate but also in some cases it can present with local complications, such as tumor necrosis, active hemorrhage, and infections. An HCC that presents as a liver abscess is usually associated with an infection formed in the liquefied necrosis of the tumor interior [6]. With only a few cases reported in the literature, we present the case of a 46-year-old male patient with a previous diagnosis of a Barcelona clinic liver cancer-stage C (BCLC-C) unresectable HCC who presented to the emergency room (ER) with right upper quadrant abdominal pain, unintentional weight loss, diaphoresis, and fever in a first-level private surgical center in Mexico City. This case is presented in order to further expand the knowledge about necrotic HCC and abscesses secondary to this, as well as highlighting the importance of an early diagnostic approach that enabled a timely therapeutic intervention.

## Case presentation

**Figure 1:**
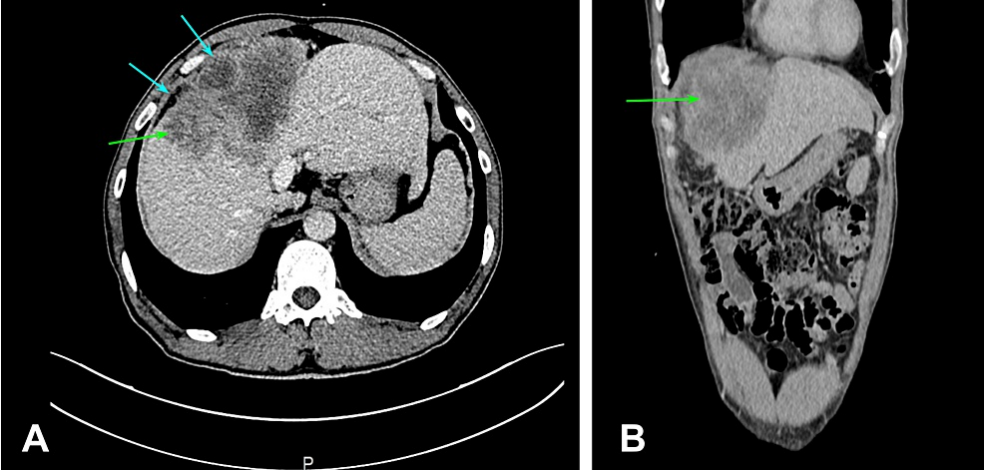
Abdominal CT scan with IV contrast. Liver presented with discretely lobulated contours in some segments, with a heterogeneous appearance at the expense of a neoformation (green arrows) that involves segments IV, VIII and part of segment VII, measuring 13 x 8.6 cm and showing reinforcement after the administration of IV contrast. Images compatible with peritumoral collections were observed, probably derived from tumor necrosis (blue arrows). A: Transverse cut. B: Coronal cut. IV: Intravenous

A 46-year-old Asian male presented to the emergency department with abdominal pain in the right upper quadrant 9/10 without pain irradiation, involuntary weight loss of 6-7 kg in the last two months, diaphoresis and fever of up to 39.0°C, predominantly in the afternoon, with transient relief after administration of an antipyretic, for a month with an increase in the last few days. As per relevant medical history, she has a diagnosis of cirrhosis secondary to chronic HBV infection (Child-Pugh A, MELD-Na 7) and unresectable poorly differentiated hepatocellular carcinoma with extensive necrosis, classified as BCLC-C as the patient presented with a lymphovascular invasion and presented Child-Pugh A. Because of this, he was in treatment with atezolizumab and bevacizumab. Due to his history, it was decided to perform an abdominal computed tomography (CT) with intravenous (IV) contrast, which reported a liver with lobulated contours in some segments, with heterogeneous appearance at the expense of a neoformation involving segments IV, VIII and part of segment VII, measuring 13 x 8.6 cm and showing reinforcement after IV contrast administration, tumoral volume 467 cc, showing images compatible with intra and peritumoral abscesses, probably derived from tumor necrosis. Also, an increase in the craniocaudal diameter of the spleen measuring 13 cm was reported and a 21 mm left para-aortic adenopathy in intimate contact with the left adrenal gland was present. Finally, a prominent 14 mm portal vein with collateral venous circulation with gastroesophageal and gastrosplenic varices was reported. The case was discussed with interventional radiology who placed a Dawson Mueller catheter in the peritumoral abscess which drained pus and residual hematic material, with partial improvement of the clinical picture (Figure 1).

**Figure 2:**
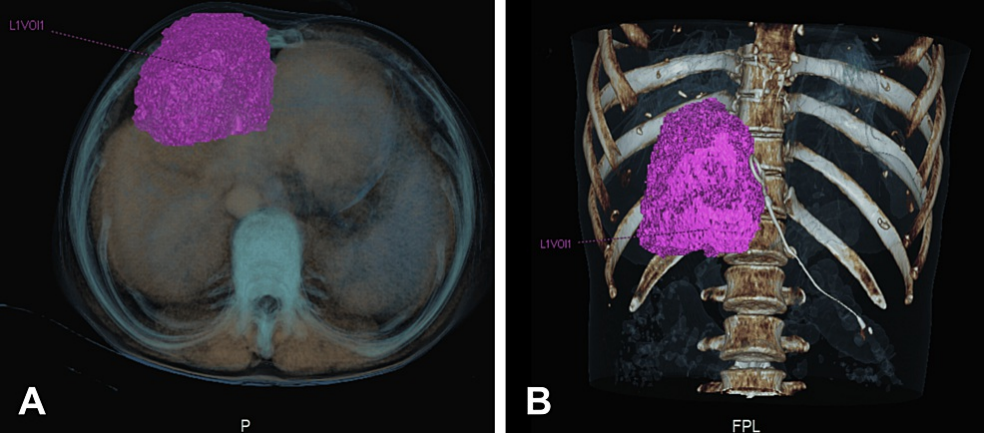
Abdominal CT three-dimensional reconstruction. Hypodense liver mass inside the HCC exhibiting an augmented peripheral rim or capsule (pink mass marked as L1VOI1). A: Transverse cut. B: Coronal cut. HCC: Hepatocellular carcinoma

Using the same CT scan previously taken, a three-dimensional reconstruction was generated. This examination clearly delineated a hypodense mass that presented an enlarged peripheral border or capsule and was practically isolated from the liver, which facilitated the precise delimitation of its limits (Figure 2).

**Figure 3:**
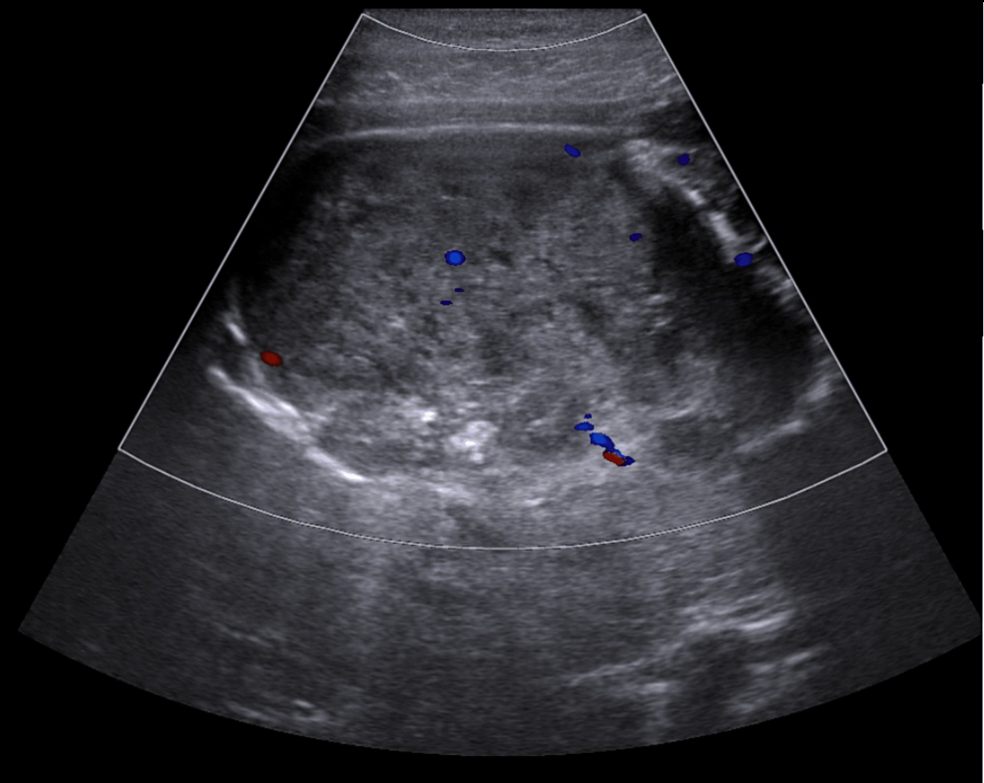
Abdominal US. Hypoechoic mass with irregular edges associated with peripheral inflammation within the HCC. HCC: Hepatocellular carcinoma

According to the diagnostic suspicion on CT, it was decided to perform a liver and biliary tract US where a hypervascular hypoechoic mass with irregular borders associated with peripheral inflammation within the HCC was reported. This mass was located in the same anatomical region as the one observed in the three-dimensional CT reconstruction. The final conclusion of the radiological report was that it was an HCC tumor with extensive necrosis and intratumoral abscess (Figure 3).

To complement the diagnostic approach, a viral hepatitis profile was requested with positive HBSAg Anti-HBCore, tumor markers with alpha fetoprotein (AFP) of 0.92 ng/dL and Ca 19-9 of 6.5 ng/dL. The tumor was unresectable according to the Milan Criteria, so it was decided to keep the patient on treatment with atezolizumab and bevacizumab, intravenous analgesic treatment with Tramadol 100 mg every 12 hours, and paracetamol 1 gram every eight hours. Due to the clear symptoms of intra-abdominal sepsis presented by the patient, it was decided to start empirical antibiotic treatment with meropenem 1 gram IV every eight hours and Linezolid 600 mg IV every 12 hours. Days later, the patient presented new fever peaks of up to 39.0ºC and started to present hematic drainage from the drain placed in the interventionist procedure. New laboratory exams were taken, which reported elevated leukocytosis at the expense of neutrophilia, elevation of gamma-glutamyl transferase (GGT), elevation of glutamic pyruvic transaminase (GPT), elevation of C-reactive protein (CRP), hyperglycemia, hypoalbuminemia, and slightly elevated urea, with no other alteration presented (Table 1).

**Table 1:** Laboratory exams. Complete blood count (CBC), relevant data from blood chemistry (BC) and hepatic profile.

Parameter	Value	Reference values
Hemoglobin	12.6 g/dL	12 - 18 g/dL
Hematocrit	38.3 %	36 - 48%
Platelets	344 x 10^3^/uL	150 - 450 x 10^3^/uL
Leukocyte Count	12.4 x 10^3^/uL	4.5 - 11 x 10^3^/uL
Absolute Neutrophils	10.7 x 10^3^/uL	2.5 - 7 x 10^3^/uL
Serum Glucose	120.9 mg/dL	70 - 100 mg/dL
BUN	14.4 mg/dL	7 - 20 mg/dL
Urea	30.8 mg/dL	5 - 20 mg/dL
Serum Creatinine	0.75 mg/dL	0.6 - 1.1 mg/dL
Serum Sodium	139.2 mEq/L	135 - 145 mEq/L
Serum Potassium	4.35 mEq/L	3.6 - 5.2 mEq/L
Serum Chlorum	100.7 mEq/L	96 - 106 mEq/L
Serum Calcium	9.2 mg/dL	8.5 - 10.5 mg/dL
Serum Phosphorus	4.4 mg/dL	2.8 - 4.5 mg/dL
Serum Magnesium	2.29 mg/dL	1.7 - 2.2 mg/dL
Total serum proteins	6.0 g/dL	6 - 8 g/dL
Albumin	2.25 g/dL	3.4 - 5.4 g/dL
Total Bilirubin	1.01 mg/dL	1 - 1.2 mg/dL
Indirect Bilirubin	0.69 mg/dL	0.2 - 1.2 mg/dL
Direct Bilirubin	0.32 mg/dL	0 - 0.35 mg/dL
Glutamic Pyruvic Transaminase (GPT)	48 U/L	4 - 36 U/L
Glutamic-Oxaloacetic Transaminase (GOT)	27 U/L	5 - 40 U/L
C-Reactive Protein (CRP)	228 mg/dL	<0.3 mg/dL

**Figure 4:**
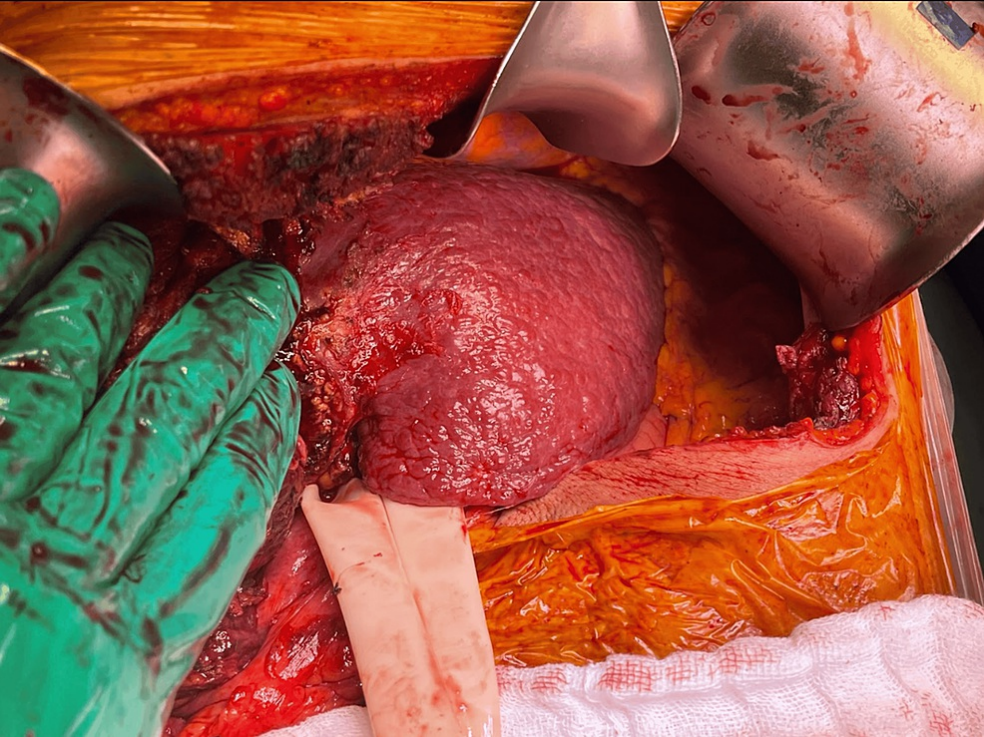
Nodular liver seen in surgery. Nodular liver with micro and macro nodules was presented, this was associated with the previous diagnosis of cirrhosis secondary to HBV and HCC HBV: Hepatitis B; HCC: hepatocellular carcinoma

Due to the clinical worsening of the patient, the characteristics of the bloody and purulent drainage, the laboratory results, and the impossibility of performing a liver transplant due to the presence of extrahepatic disease and HBV infection, the need for early salvage surgical treatment was determined. Therefore, it was decided to perform an exploratory laparotomy with a Chevron incision. After the incision, a hepatic surface with micro and macro nodules was observed, associated with the previous diagnosis of cirrhosis secondary to HBV and HCC (Figure 4).

**Figure 5:**
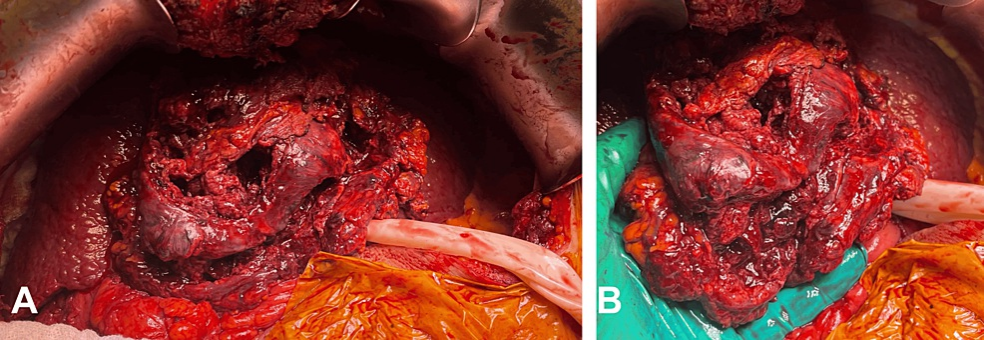
Extensive necrosis and intra- and peritumoral abscess. Extensive necrosis and intra- and peritumoral abscess with extension to segments V and VII with active intraluminal bleeding.

Open hepatic necrosectomy of the exophytic tumor of 15 x 10 cm with necrosis and residual abscess which presented active intratumoral venous bleeding (Figure 5).

**Figure 6:**
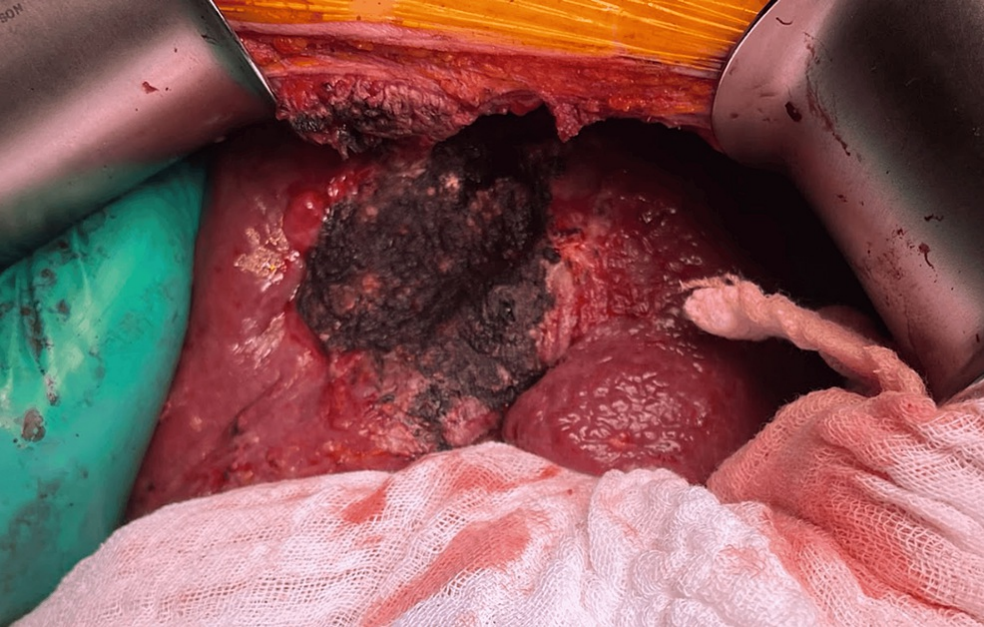
Non-anatomical hepatectomy. Remnant liver after non-anatomical hepatectomy of segments IV, V, and VII.

**Figure 7:**
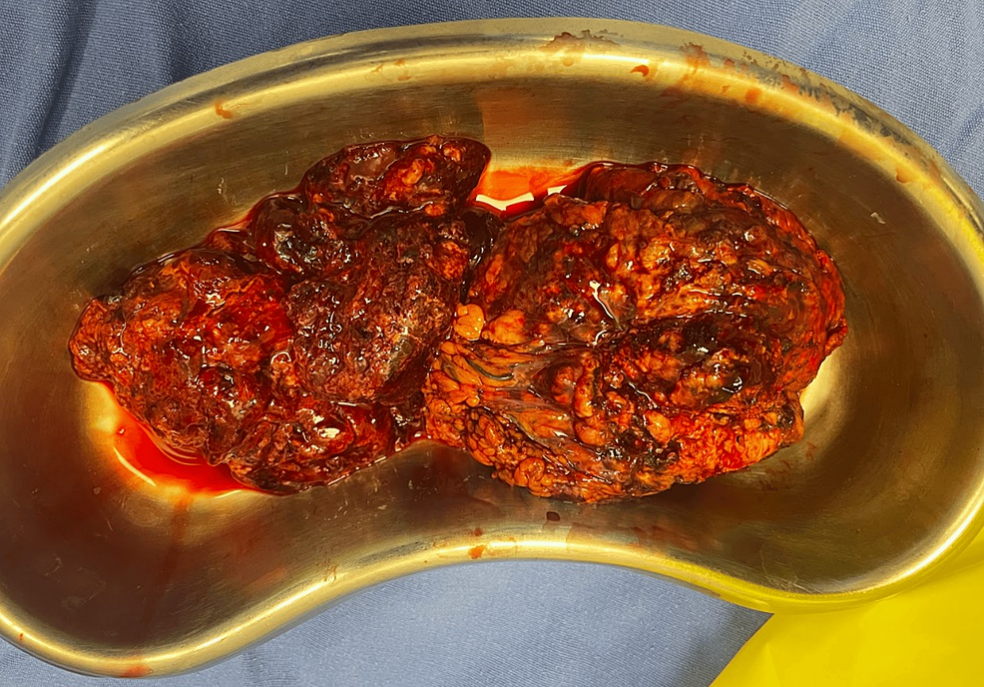
Anatomical piece sent for histopathological report. Exophytic liver tumor dependent on liver segment IV of approximately 10 x 15 cm.

Likewise, a non-anatomic hepatectomy of segments IV, V and VII guided by transoperative US with exploration of the right biliary tract was performed, the liver transection was realized with Ultrasonic device and monopolar energy (Figure 6), in addition to exploration of the right bile duct, partial resection of the anterior abdominal wall due to tumor infiltration + standard portal and perihilar lymphadenectomy, lavage and drainage of the abdominal cavity with 0 saline solution. 9% saline and sodium hypochlorite, with placement of Jackson-Pratt drainage to the hepatic surgical bed. Besides that, the final anatomical piece was sent for pathology and cultures (Figure 7).

**Figure 8:**
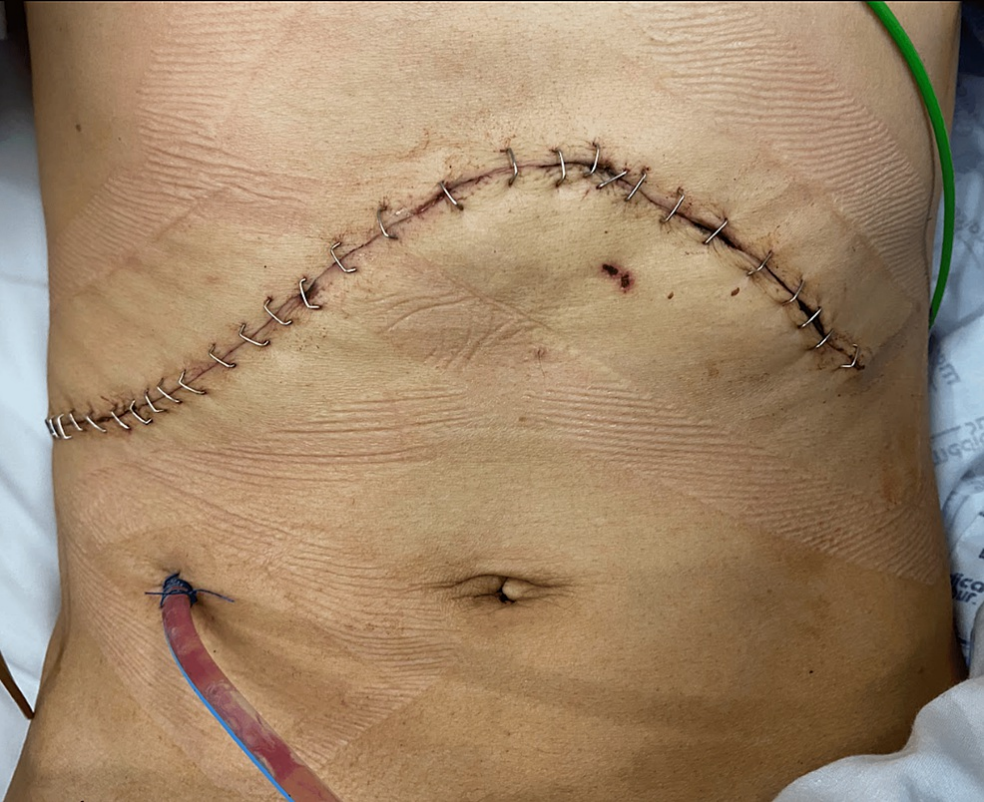
Abdominal wall closure. Primary closure in layers and skin closure using staples, Jackson-Pratt drain located below skin closure on the right lower quadrant of the abdomen.

Finally, closure of the abdominal wall was carried out without complications, presenting a primary closure in layers with polypropylene and skin closure using staples (Figure 8). No intraoperative blood products were transfused, perioperative hemodynamic stability was maintained and the estimated blood loss was 500 mL. Liver hemostasis was performed with monopolar, silk, and argon gas. After surgery, the patient remained under postoperative surveillance in the Intensive Care Unit (ICU), where he continued to improve, presented afebrile for the whole time during hospitalization had partial analgesic control with the previously mentioned analgesic scheme, and was finally discharged from the hospital after one week in ICU and one week in regular hospitalization.

## Discussion

As previously mentioned, HCC is one of the most common cancer-related deaths around the world. It presents as the most common primary liver cancer, with more than 80% of all hepatic cancers being diagnosed as HCC. Also, it is important to mention that almost 85% of all cases are estimated to occur in developing countries, such as the one in this case. At the same time, the principal risk factor is chronic infection from HBV and HCV, as these account for 80% of all cases around the world. Another prevalent cause is NAFLD, currently up to 20% of all cases are attributed to this cause. Alcohol cirrhosis is the second most common cause of HCC [7]. Another important point to mention is the existence of different patterns of carcinogenesis, being the most common mutations in the TERT promoter, present in 65% of all cases [8]. This is why, many preventable measures can be taken in order to prevent the formation of HCC, this is also known as primary prevention strategies such as a healthy diet, diabetes mellitus prevention, HBV vaccination, and avoiding alcohol intake. Worldwide, multiple perspectives exist as a secondary prevention of HCC, principally in patients who have been diagnosed with HCC. These strategies are focused on looking for early tumor detection and thus, allowing appropriate early management. According to new guidelines published by the European Association for the Study of the Liver (EASL) all people with cirrhosis and advanced fibrosis, from F3, regardless of its etiology need to undergo surveillance [9].

Many modalities to perform HCC surveillance exist; however, EASL guidelines recommend abdominal ultrasound (US) as data shown in their studies show that in terms of cost-effectiveness, abdominal US is the best image modality. Also, some clinicians use AFP levels, however, this is not endorsed by EASL as part of its surveillance protocol due to the high rate of false-negative test results [9]. It is important to emphasize that this only applies to surveillance because when discussing diagnostic modalities, other image studies are preferred over abdominal US. This is because as opposed to most solid cancers, hepatic tumor diagnosis relies on imaging studies as no histopathological confirmation is required nor encouraged among clinicians. The diagnosis of HCC is made through the use of an abdominal CT scan or magnetic resonance imaging (MRI) by using an IV contrast and observing the arterial and venous phases. The classic presentation of an HCC is a non-rim arterial phase hyperenhancement followed by washout during the portal venous phase [10]. As previously mentioned, the biomarker AFP is also used during the diagnostic approach. This is a biomarker that, beyond serving as a diagnostic tool, has a monitoring function in patients who already have a radiological diagnosis of HCC. The use of it as a diagnostic tool has not been widely accepted as sex (being a woman), presence of the smoking habit, presence of viral etiology, vascular invasion, or extrahepatic spread have been associated with variations in serum levels, resulting in wide variations that limit its diagnostic function [11].

In addition to being a cancer with a high mortality rate, the morbidity associated with HCC also presents a challenge in these patients. This is usually associated with complications that can occur locally in the liver secondary to anatomical and histopathological changes secondary to HCC, such as liver failure, hemorrhage, necrosis, and local infections that when complicated can lead to peritonitis and sepsis. Tumor necrosis is usually associated with relative hypoperfusion of the fast-growing tissue inside the tumor, so it is frequently observed in many histopathological pieces of solid tumors. On the other side, HCC is traditionally known for being a highly vascularized cancer so it is believed that necrosis in these cases is associated with an aggressive tumor growth that results in impaired oxygen delivery as well as local inflammation in the microenvironment [12]. As presented in this case, patients with hepatic abscesses present with a fever, right upper quadrant pain, leukocytosis, neutrophilia, hypoalbuminemia, and elevated hepatic transaminases, mostly alkaline phosphatase levels. For an abscess to present there must be a coexisting tumor necrosis. The US image is the choice diagnostic tool and it will present as a low/echoic to mixed/echoic lesion with blurred edges associated with inflammation in the surrounding area [6]. 

In addition to the suggestive US image, one of the next criteria should be met: positive blood culture for bacteria, aspirated material being purulent or having a positive culture or presenting fevers higher than 38.5°C for more than five days and a leukocyte count higher than 12 103/uL without any other apparent cause [13]. Management of the abscess is preferred to be done through percutaneous drainage [14]. This is always accompanied by antibiotic therapy; however, it is important to mention that only a few publications in literature establish a correct course of treatment, and individualization of every case is always encouraged by guides [13]. Liver necrosectomy should be encouraged in cases where the necrosis is extensive and superinfection is present or in cases where the patient reports clear symptoms of sepsis, as in the case presented here. The importance of recognizing the clinical picture and using paraclinical studies to reach the diagnosis of extensive and superinfected necrosis is of vital importance since this can change the morbidity associated with HCC in a drastic way [15].

## Conclusions

It is a very rare presentation of an unresectable poorly differentiated hepatocellular carcinoma with extensive necrosis with peri- and intra-tumor abscesses, which caused severe multi-treated abdominal sepsis in a Barcelona C and Child-Pugh B cirrhotic patient, which required surgical resection. A radical and aggressive emergency surgical treatment was the best management for the infectious and hemorrhagic complications presented. It is vitally important to highlight the importance of using different modalities of imaging studies in order to reach an accurate diagnosis in the shortest time possible. The fact of having used not only CT with IV contrast along with its three-dimensional reconstructions but also having complemented it with an abdominal US allowed us to find the diagnosis of extensive tumor necrosis with superinfection. Based on the correct diagnosis, the best surgical management was determined, which in this case was a necrosectomy. Likewise, this case reinforces the literature already published worldwide by showing a classic case, associated with the main risk factors and complications associated with HCC.
